# HBV mutations in EnhII/BCP/PC region contribute to the prognosis of hepatocellular carcinoma

**DOI:** 10.1002/cam4.2169

**Published:** 2019-04-29

**Authors:** Zijun Ge, Ting Tian, Lijuan Meng, Ci Song, Chengxiao Yu, Xin Xu, Jibin Liu, Juncheng Dai, Zhibin Hu

**Affiliations:** ^1^ State Key Laboratory of Reproductive Medicine Nanjing Medical University Nanjing China; ^2^ Department of Epidemiology, Center for Global Health, School of Public Health Nanjing Medical University Nanjing China; ^3^ Jiangsu Key Lab of Cancer Biomarkers, Prevention and Treatment, Collaborative Innovation Center for Cancer Medicine Nanjing Medical University Nanjing China; ^4^ Integrated Hospital of Traditional Chinese Medicine Southern Medical University Guangzhou China; ^5^ Department of Hepatobiliary Surgery Nantong Tumor Hospital Nantong China

**Keywords:** HBV genotype, HBV mutation, HCC

## Abstract

**Background:**

Accompanied by HBV infection, HBV mutations gradually occur because HBV polymerase appears proofread deficiencies. In our previous study, we have identified that EnhII/BCP/PC mutations and genotype C of HBV DNA were associated with hepatocellular carcinoma (HCC) risk. In this study, we extend our research to explore HCC prognosis associated genotype and mutations in EnhII/BCP/PC regions.

**Methods:**

We designed a case‐cohort study of 331 HCC patients to evaluate the effects of the HBV genotypes and mutations on HCC survival. Log‐rank test and Cox proportional hazard models were used for the analyses.

**Results:**

Results showed that genotype C, which was more frequent in HBV‐related HCC (77.4%), presented a negative signal with HCC survival. Interestingly, we detected a significant association between EnhII/BCP/PC mutation nt1753 and HCC prognosis (Log‐rank *P* = 0.034). Subgroup analysis revealed that this risk effect was more pronounced in non‐B genotype (*P* = 0.090 for heterogeneity test). We also detected a borderline multiplicative interaction between genotypes of nt1753 and HBV genotype on HCC survival (*P* for interaction = 0.069).

**Conclusions:**

These findings indicated that, in Chinese population, nt1753 in EnhII/BCP/PC region might be a novel marker for HCC prognosis.

## 1 INTRODUCTION

Liver cancer ranged as the seventh most common cancer and the fourth leading cause of global cancer deaths (http://gco.iarc.fr/today/home). It was estimated that the number of the new liver cancer cases per year was 554,000 for men and 228,000 for women, respectively, over 50% of which occurred in China.^1^ Hepatocellular carcinoma (HCC), as the most important pathological of liver cancer, has a poor prognosis (with a poor 5‐year survival rate of about 7% after treatment).^2^, ^3^ Multiple risk factors were involved in the HCC prognosis, including tumor size, tumor quantity, degree of inflammation, and hepatitis virus (HBV), etc.^4^, ^5^, ^6^ Among them, HBV attributed to be the major reason for HCC in China.^7^ Nowadays, the research on HCC is inseparable from the discussion of HBV.

So far, some studies had focused on the associations between HBV genotypes, key mutations, and HCC prognosis. The determination of the HBV genotype is defined based on whether the difference between the groups of the complete genomic sequence exceeds 8%.^8^, ^9^, ^10^ The effects for HCC risk have been certified endemic in China.^11^ Moreover, the HBV genotypes have also been proved to implicate with other liver diseases and influence on interferon treatment.^12^, ^13^, ^14^ On the other hand, HBV precore mutations for the HBV‐related HCC (HBV‐HCC) cancer risk have been well studied in recent years.^15^, ^16^, ^17^ The A1762T/G1764A mutations of basal core promoter (BCP) and PreC/C nt1915, nt1896 mutational sites independently impact the postoperative survival of HCC patients.^18^, ^19^, ^20^ However, whether HBV genotypes taking together with mutations impact the HCC prognosis is still confusing.^21^ As a continuity of our previous study,^15^ which has identified HBV genotype C and EnhII/BCP/PC mutations were associated with HCC risk, we recruited 331 HCC patients as a case‐cohort to evaluate the prognostic effects of HBV genotypes and EnhII/BCP/PC mutations in a Chinese Population.

## 2 PATIENTS AND METHODS

### 2.1 Patients

The patients were reported in our previous study.^22^ Briefly, we recruited 414 HCC patients from the First Affiliated Hospital of Nanjing Medical University and Nantong Tumor Hospital and in China. Date of patients’ enrollment was defined as baseline‐time. The date of death or the last follow‐up was defined as outcome time. During the follow‐up, we prospectively telephone followed up all patients every 3 months. Patients who smoked one cigarette per day for over one year were defined as smokers, and those who consumed one or more alcohol drinks a week for over 6 months were categorized as alcohol drinkers. Taking account of population heterogeneity: (1) patients who had undergone tumor surgery were excluded in our study; (2) patients with Barcelona Clinic Liver Cancer (BCLC) classification B and C grade were included.^23^ Finally, 331 HCC patients who had completed follow‐ups and baseline information were included in this study. The final response rate was 80.0%. Age ranged from 21 to 89, with 284 males and 47 Females. The median survival time was 14.5 months.

### 2.2 Serological HBV and HCV detecting

We use the platform of enzyme linked immunosorbent assay to detect serum HBsAg and anti‐HCV of all the patients (Kehua, Shanghai, China).

### 2.3 Detection of HBV genotypes and subgenotypes

Using the High Pure Viral Nucleic Acid kits (Roche, Mannheim, Germany), we extracted virus nucleic acid from 200 L serum of the included patients. Nested‐PCR was then used to amplify target genome.^15^ In the first round PCR, common primer P1‐S (TTTGCGGGTCACCATATTCTTGG) and P1‐AS (CGAACCACTGAACAAATGGCACTAG) was used to enrich region including genotype A, B, C, D. Genotype‐specific primers were then used to amplify genotype A, B, C, D, the detailed primer sequence were listed in our previous study.^15^ All of the experiments were blindly performed. PCR assays of different samples were added into single PCR tubes. The success rates of genotypes were over 90%.

### 2.4 HBV mutation detection

EnhII/BCP/PC region was amplified by nested‐PCR. The primers of the nested‐PCR were listed in our previous study.^15^ The amplified products were then purified and sequenced by using ABI PRISM BigDye sequencing kits and an ABI 3730 Genetic Analyser (Applied Biosystems, CA). The sequences were aligned and analyzed using MEGA 6.0 software. Wild type at each site was defined as the nucleotide with the highest frequency among the HBV persistent carriers. Nucleotide substitutions with the other three nucleotides or indels were defined as mutations. A site with combined SNV frequencies >10% was defined as a hotspot.

### 2.5 Statistical analysis

Median survival time (MST) was summarized to describe the time‐to‐event data, otherwise mean survival time was presented. Kaplan‐Meier method was used to estimate survival. The association between MST and demographic characteristics, clinical features and different genotypes was estimated by log‐rank test. In the analysis of risk factors, univariate and multivariable Cox proportional hazard regression models were performed to estimate the hazard ratio (HR) for HCC, adjusted for age at baseline, gender (male and female), smoking status (no or yes), drinking status (no or yes), BCLC stage (stage B and C), and chemotherapy or TACE (transcatheter hepatic arterial chemoembolization) status (none or yes). To determine the predictors of HCC prognosis, we further performed Stepwise Cox regression model, and set a logically significant level with *P* < 0.050 for entering and *P* > 0.050 for removing. The heterogeneity between subgroups was analyzed with the Chi‐square‐based Q test. We used Statistical Analysis System software (version 9.1.3; SAS Institute, Cary, NC). All tests were two‐sided and the statistical significance level was set at *P* < 0.05.

## 3 RESULTS

The characteristics and clinical features of all patients were described previously.^22^ In brief, patients’ survival time was significantly influenced by drinking and chemotherapy or TACE status. As seen in Table 1, the frequency of genotype C was higher in never‐drinker and stage C patients than genotype B (*P* = 0.017, 0.011 in different drinking status and BCLC stage, respectively).

**Table 1:** Characteristics of patients at listing according to HBV genotypes

Variables	HBV genotypes			*P* value
	B	BC	C	
Age				
≤53	23 (14.6)	13 (8.3)	121 (77.1)	0.991
>53	21 (14.2)	12 (8.1)	115 (77.7)	
Gender				
Male	39 (14.9)	24 (9.2)	198 (75.9)	0.147
Female	5 (11.4)	1 (2.3)	38 (86.4)	
Smoking status				
No	11 (9.8)	7 (6.3)	94 (83.9)	0.101
Yes	33 (17.1)	18 (9.3)	142 (73.6)	
Drinking status				
No	11 (9.2)	6 (5.0)	102 (85.7)	0.017
Yes	33 (17.7)	19 (10.2)	134 (72.0)	
BCLC stage				
Stage B	44 (15.8)	23 (8.3)	211 (75.9)	0.011
Stage C	0 (0)	2 (7.4)	25 (92.6)	
Chemotherapy or TACE				
None	11 (12.6)	11 (12.6)	65 (74.7)	0.213
Yes	33 (15.1)	14 (6.4)	171 (78.4)	
All patients	44 (14.4)	25 (8.2)	236 (77.4)	

Abbreviations: BCLC, Barcelona Clinic Liver Cancer stage; CI, confidence intervals; HR, hazard ratio; TACE, transcatheter hepatic arterial chemoembolization.

**Figure 1:**
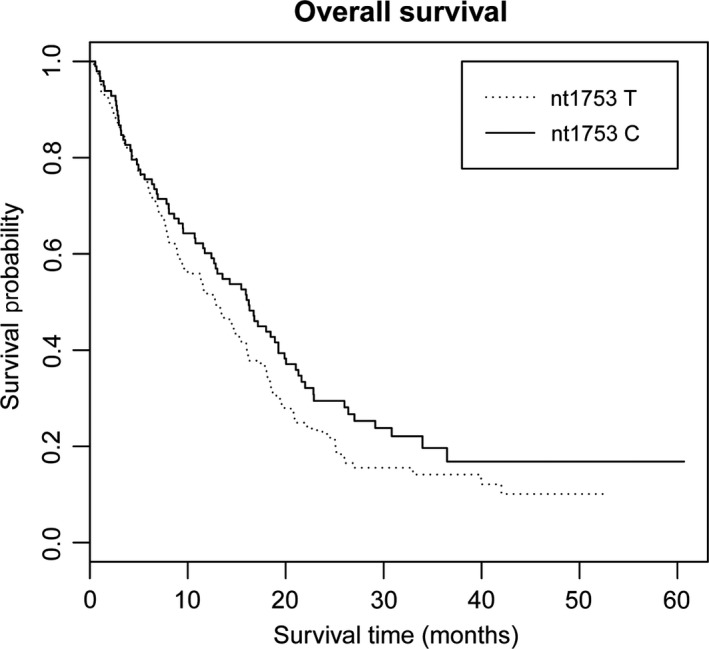
Kaplan‐Meier plots of HCC patients’ overall survival by nt1753 mutation, log‐rank *P* = 0.034. X‐axis, HCC patients’ survival time (months); Y‐axis, HCC patients’ survival probability

There was no significant association between HBV genotypes and HCC survival (Table 2). Multivariate Cox regression model was further applied to select HBV mutations which associated with HCC survival, with adjustment––for age, sex, drinking and smoking status, chemotherapy or TACE, and BCLC stage. Mutation nt1753 was associated with HCC prognosis (Log‐rank *P* = 0.034; HR = 0.73, 95% CI = 0.55‐0.97, *P* = 0.035) (Table 3). Kaplan‐Meier plot of HCC patients’ overall survival by nt1753 mutation was shown in Figure 1. When we used multivariate stepwise Cox regression analysis, nt1753 sustained in the last predictive model together with age, drinking status, and therapy status (HR = 0.72, 95% CI = 0.56‐0.93, *P* = 0.013) (Table 4).

**Table 2:** HBV genotype frequencies and HCC survival

HBV genotype	HCC Patients	Deaths	MST	Log‐rank	Crude HR	*P* value	Adjusted HR	*P* value^a^
	(n = 305) N (%)	(n = 238) N (%)	(mo)	*P* value	(95% CI)		(95% CI)^a^	
Model 1								
B	44 (14.43)	34 (11.15)	13.54		1	—	1	—
BC	25 (8.20)	20 (6.56)	14.29	0.644	1.11 (0.64‐1.93)	0.705	0.74 (0.42‐1.31)	0.298
C	236 (77.38)	184 (60.33)	14.69	0.617	0.91 (0.63‐1.32)	0.620	0.86 (0.59‐1.25)	0.423
Model 2								
B related	69 (22.62)	54 (17.70)	14.29		1	—	1	—
Non‐B	236 (77.38)	184 (60.33)	14.69	0.398	0.88 (0.65‐1.19)	0.399	0.97 (0.71‐1.33)	0.837

Note

B related genotypes including B, BC. Non‐B genotypes including C, D. Abbreviation: HCC, hepatocellular carcinoma.

a Cox proportional hazard regression analyses adjusted for age, gender, smoke, drink, BCLC stage, and chemotherapy or TACE.

**Table 3:** The frequencies of the nucleotide substitutions in the EnhII/BCP/PC region of HBV in the HCC patients and HCC survival

Substitutions	HCC Patients	Deaths	MST	Log‐rank	Crude HR	*P *value	Adjusted HR^a^	*P* value^a^
	N (%)	N (%)	(mo)	*P *value	(95% CI)		(95% CI)^a^	
C1653T								
C	201 (74.2)	160 (79.6)	14.69	—	1	—	1	—
T	70 (25.8)	52 (74.3)	10.71	0.868	1.03 (0.75‐1.41)	0.868	0.93 (0.67‐1.27)	0.635
C1673T								
C	229 (84.5)	178 (77.7)	13.54	—	1	—	1	—
T	42 (15.5)	34 (81.0)	13.54	0.495	1.14 (0.79‐1.64)	0.496	1.22 (0.84‐1.78)	0.293
T1674C/G								
T	193 (71.2)	151 (55.7)	13.34	—	1	—	1	—
C/G	78 (28.8)	61 (22.5)	16.00	0.659	0.94 (0.69‐1.26)	0.660	0.84 (0.62‐1.14)	0.255
A1703G								
A	231 (85.2)	178 (65.7)	13.54	—	1	—	1	—
G	40 (14.8)	34 (12.6)	14.29	0.753	1.06 (0.73‐1.53)	0.753	1.05 (0.72‐1.52)	0.814
G1719T								
G	88 (32.5)	70 (25.8)	14.49	—	1	—	1	—
T	183 (67.5)	142 (52.4)	12.85	0.603	0.93 (0.70‐1.24)	0.604	0.87 (0.65‐1.17)	0.353
A1726C								
A	233 (86.0)	184 (67.9)	13.54	—	1	—	1	—
C	38 (14.0)	28 (10.3)	13.54	0.860	1.04 (0.70‐1.54)	0.860	0.99 (0.66‐1.48)	0.954
T1727A/G								
T	42 (15.5)	32 (76.2)	13.54	—	1	—	1	—
A/G	229 (84.5)	180 (78.6)	13.54	0.556	0.89 (0.61‐1.30)	0.557	0.94 (0.64‐1.38)	0.752
C1730G								
C	229 (84.5)	181 (79.0)	13.44	—	1	—	1	—
G	42 (15.5)	31 (73.8)	14.49	0.778	1.06 (0.72‐1.55)	0.778	1.02 (0.69‐1.51)	0.904
A1752G								
A	242 (89.3)	191 (78.9)	13.44	—	1	—	1	—
G	29 (10.7)	21 (72.4)	15.47	0.872	0.96 (0.61‐1.51)	0.872	1.06 (0.67‐1.67)	0.809
T1753C								
T	176 (64.9)	142 (80.7)	12.58	—	1	—	1	—
C	95 (35.1)	70 (73.7)	16.30	0.034	0.73 (0.55‐0.97)	0.035	0.75 (0.56‐1.01)	0.059
A1762T								
A	51 (18.8)	39 (76.5)	14.49	—	1	—	1	—
T	220 (81.2)	173 (78.6)	13.54	0.765	1.05 (0.74‐1.49)	0.766	1.05 (0.74‐1.49)	0.789
G1764A								
G	33 (12.2)	23 (69.7)	14.46	—	1	—	1	—
A	238 (87.8)	189 (79.4)	13.54	0.700	1.09 (0.71‐1.68)	0.701	1.07 (0.69‐1.66)	0.757
A1762T/G1764A								
A/G	32 (9.7)	22 (73.9)	14.46	—	1	—	1	—
T/A	299 (72.2)	236 (79.5)	14.29	0.699	1.09 (0.70‐1.69)	0.699	1.08 (0.70‐1.68)	0.723
G1799C								
G	48 (17.7)	36 (75.0)	14.69	—	1	—	1	—
C	223 (82.3)	176 (78.9)	12.85	0.983	1.00 (0.70‐1.44)	0.983	1.15 (0.80‐1.66)	0.457
A1846T								
A	172 (63.5)	139 (80.8)	13.34	—	1	—	1	—
T	99 (36.5)	73 (73.7)	14.46	0.585	0.92 (0.70‐1.23)	0.585	0.95 (0.71‐1.27)	0.728
G1896A								
G	119 (43.9)	94 (79.0)	15.47	—	1	—	1	—
A	152 (56.1)	118 (77.6)	13.01	0.245	1.17 (0.89‐1.54)	0.246	1.23 (0.94‐1.63)	0.137
G1899A								
G	187 (69.00)	152 (81.28)	12.85	—	1	—	1	—
A	84 (31.00)	60 (71.43)	16.10	0.067	0.76 (0.56‐1.02)	0.068	0.69 (0.51‐0.95)	0.020
G1915A/C								
G	229 (84.5)	177 (77.3)	14.65	—	1		1	
A/C	42 (15.5)	35 (83.3)	11.56	0.653	1.09 (0.76‐1.56)	0.654	1.44 (0.98‐2.10)	0.061
C1969T								
C	234 (86.4)	181 (77.4)	13.70	—	1		1	
T	37 (13.7)	31 (83.8)	12.42	0.766	1.06 (0.72‐1.55)	0.766	1.11 (0.76‐1.64)	0.581
A1979G								
A	230 (84.9)	181 (78.7)	13.54	—	1		1	
G	41 (15.1)	31 (75.6)	14.95	0.396	0.85 (0.58‐1.24)	0.397	0.83 (0.56‐1.22)	0.341

a Cox proportional hazard regression analyses adjusted for age, gender, smoke, drink, BCLC stage, and chemotherapy or TACE.

**Table 4:** Multivariate Cox regression analysis on HCC patients’ survival

Variables	β^a^	SE^b^	HR (95% CI)	*P* value
Stepwise regression analysis				
Chemotherapy or TACE (yes vs no)	−0.7585	0.1513	0.47 (0.35‐0.63)	<0.001
Age (>53 vs ≤53)	−0.4753	0.1352	0.62 (0.48‐0.81)	<0.001
nt1753 (C vs T)	−0.3224	0.1299	0.72 (0.56‐0.93)	0.013
Drinking status (yes vs no)	0.3451	0.1283	1.41 (1.10‐1.82)	0.007

Note

*P*: Cox proportional hazard regression analyses adjusted for age, gender, smoke, drink, BCLC stage, chemotherapy or TACE and HBV genotype and mutations.

a β is the estimated parameter of the regression model.

b SE is the standard error of the regression model.

Stratification analysis was performed to evaluate the different effects of nt1753 on HCC survival, according to subgroups of demographic characteristics, BCLC stage, and therapy status (Table 5). We found the results that the effect of nt1753 was more significant in non‐B genotype (adjusted HR = 0.74, 95% CI = 0.54‐1.02 in Non‐B genotype; adjusted HR = 1.64, 95% CI = 0.69‐3.87 in B genotype; *P* = 0.090 for heterogeneity test). Multiplicative interaction analysis was then performed and slightly significant signal on HCC survival was found between nt1753 genotypes and HBV genotypes (*P* = 0.069) (Table 6). Crossover analyses revealed that taking subjects with nt1753T genotype and non‐B HBV genotype as reference, patients with nt1753C and non‐B HBV genotype had a significantly decreased hazard of death (HR = 0.73, 95% CI = 0.53‐1.01).

**Table 5:** Stratified analyses of combined effect of nt1753 associated with HCC patients’ survival

Variables	nt1753 (deaths/censor)		Adjusted HR (95% CI)^a^	*P* for heterogeneity
	T	C		
Age				
≤53	78/15	37/15	0.77 (0.50‐1.19)	0.654
>53	64/19	33/12	0.89 (0.56‐1.41)	
Gender				
Male	20/3	9/4	0.94 (0.36‐2.47)	0.967
Female	122/31	61/21	0.92 (0.66‐1.29)	
Smoking status				
No	43/15	25/10	0.92 (0.53‐1.59)	0.843
Yes	99/19	45/15	0.86 (0.59‐1.26)	
Drinking status				
No	45/17	26/11	1.02 (0.60‐1.74)	0.520
Yes	97/17	44/14	0.82 (0.55‐1.22)	
BCLC stage				
Stage B	129/31	62/23	0.76 (0.55‐1.05)	0.352
Stage C	13/3	8/2	1.33 (0.43‐4.14)	
Chemotherapy or TACE				
No	49/9	19/5	0.91 (0.50‐1.64)	0.769
Yes	93/25	51/20	0.82 (0.57‐1.18)	
HBV genotype				
B related	38/12	7/0	1.64 (0.69‐3.87)	0.090
Non‐B	98/22	64/23	0.74 (0.54‐1.02)	

Abbreviations: CI, confidence intervals; HR, hazard ratio.

a Adjusted for age, gender, smoke, drink, BCLC stage, Chemotherapy or TACE and HBV genotype.

**Table 6:** Interactive effect of nt1753 and HBV genotype associated with HCC patients’ survival

HBV genotype	nt1753	Patients	Deaths	Adjusted HR^a^ (95%CI)	*P* value^a^
		N (%)	N (%)		
Non‐B	T	123 (46.59)	101 (82.11)	1.00	—
Non‐B	C	84 (31.82)	61 (72.62)	0.73 (0.53‐1.01)	0.060
B related	T	50 (18.94)	38 (76.00)	0.85 (0.58‐1.25)	0.405
B related	C	7 (2.65)	7 (100.00)	1.40 (0.64‐3.06)	0.402
*P* for multiplicative interaction					0.069

Abbreviations: CI, confidence intervals; HCC, hepatocellular carcinoma; HR, hazard ratio.

a Adjusted for age, gender, smoke, drink, BCLC stage, Chemotherapy or TACE and HBV genotype.

## 4 DISCUSSION

In this study, we evaluated the effects of HBV genotypes and mutations in EnhII/BCP/PC region on advanced HCC patients’ survival and demonstrated that nt1753 may be a potential biomarker to predict the prognosis for patients with advanced HCC.

Our previous large‐scale multi‐center case‐control study 15 indicated that patients with genotype C were more susceptible to develop HCC. This study may provide slight evidence to support the conclusion (shown in Table 1). In addition, we found that HCC patients with genotype C were prone to suffering more malignant tumors than those with genotype B.

When compared with patients with genotype B, patients with genotype C were in connection with higher HBV load,^24^ more serious liver diseases, such as liver cirrhosis or HCC,^25^, ^26^, ^27^ had been reported in several Asian countries. The earlier spontaneous HBeAg seroconversion developed in patients with genotype B, the less progressive liver disease would occur.^28^ While evidence for genotype C correlated with poor liver disease outcomes was sounded, genotype C not a predictor for HCC patients’ overall survival (OS) in our study. Despite some studies indicated independent risk effects of genotype C for HCC tumor recurrence, we need to be careful drawing conclusions considering the small sample size.^29^, ^30^

Nt1753 located in the EnhII region (embedded in X gene) was independently associated with HCC OS, which was the most essential finding in this study. The prospective effect was more pronounced in non‐B genotype. Nt1753 mutation in the region led to the amino acid (aa) transitions at aa127 (I‐to‐T/N/S), and it has been shown to be independently and significantly associated with HCC risk in our previous multi‐center case‐control study.^15^ The opposite effect indicated that subjects with nt1753C had a greater risk for developing HCC; afterwards, the survival time of HCC patients with nt1753C were longer than that in patients with nt1753T. In a previous cohort in Taiwan, they found that the HBV e‐antigen (HBeAg)‐negative patients had a higher frequency of mutations of T1753C than HBeAg‐positive patients.^31^ Another study showed that clones with mutations at positions 1753/1762/1764 exhibited increased‐replication phenotypes.^32^ However, one cross‐sectional analysis in Chinese population found that mutation at 1753/1762/1764 and genotype was not associated with viral loads in either HBeAg or anti‐HBe positive subjects.^33^The association between HBV viral load and mutation of nt1753 was still unclear. Moreover, many studies have demonstrated that a high viral load was an adverse prognostic factor for survival of HBV‐related HCC patients.^34^, ^35^, ^36^ Then the association of T1753C mutation and HBV‐related HCC patients’ survival were still controversial. In addition, HBV exist as quasispecies, in which the mutant possesses differing levels of fitness in a range of environments,^37^ from chronic hepatitis to advanced HCC. In order to verify the association between nt1753 mutation and HCC survival, large sample population studies or functional experiments are needed in the future.

Surprisingly, the effect of HBV genotypes and mutations on HCC prognosis has not been examined by systematically dissecting EnhII/BCP/PC region. What's more, when compared to previous studies, the successful rates of detecting HBV genotypes, subgenotypes, and mutations were more reasonable. However, our study has several limitations as well: moderate sample size needs to increase to ensure the stability of the results; in addition, the HCC patients were followed up by telephone in this study, the cirrhosis diagnosis and the antiviral treatment scheme was not obtained. Therefore, we did not show HBV DNA, ALT and AST level, cirrhosis status in this study; otherwise, external validation with independent population may further solid the findings.

## 5 CONCLUSIONS

In conclusion, our study has demonstrated nt1753C in EnhII/BCP/PC region might be an independent predictors for HCC prognosis. Further large population study and functional experiments were needed to polish the conclusion.

## ETHICS APPROVAL AND CONSENT TO PARTICIPATE

This study was approved by the institutional review board of Nanjing Medical University. All the patients provide their written informed consent to participate in this study and the ethics committees approved this consent procedure.

## CONFLICT OF INTERESTS

The authors declare that they have no competing interests.

## References

[1] Richman DM , Tirumani SH , Hornick JL , et al. Beyond gastric adenocarcinoma: multimodality assessment of common and uncommon gastric neoplasms. Abdom Radiol (NY). 2017;42(1):124‐140. 27645897 10.1007/s00261-016-0901-x PMC5247350

[2] Carr BI . Hepatocellular carcinoma: current management and future trends. Gastroenterology. 2004;127(5 Suppl 1):S218‐S224. 15508087 10.1053/j.gastro.2004.09.036

[3] Merion RM . Current status and future of liver transplantation. Semin Liver Dis. 2010;30(4):411‐421. 20960380 10.1055/s-0030-1267541

[4] Maki A , Kono H , Gupta M , et al. Predictive power of biomarkers of oxidative stress and inflammation in patients with hepatitis C virus‐associated hepatocellular carcinoma. Ann Surg Oncol. 2007;14(3):1182‐1190. 17195915 10.1245/s10434-006-9049-1

[5] Okada S , Shimada K , Yamamoto J , et al. Predictive factors for postoperative recurrence of hepatocellular carcinoma. Gastroenterology. 1994;106(6):1618‐1624. 8194710 10.1016/0016-5085(94)90419-7

[6] Wang C , Zhang F , Fan H , et al. Sequence polymorphisms of mitochondrial D‐loop and hepatocellular carcinoma outcome. Biochem Biophys Res Comm. 2011;406(3):493‐496. 21345333 10.1016/j.bbrc.2011.02.088

[7] Parkin DM , Bray F , Ferlay J , Pisani P . Global Cancer Statistics, 2002. CA Cancer J Clin. 2005;55(2):74–108. 15761078 10.3322/canjclin.55.2.74

[8] Norder H , Courouce AM , Magnius LO . Molecular basis of hepatitis B virus serotype variations within the four major subtypes. J Gen Virol. 1992;73(Pt 12):3141‐3145. 1469353 10.1099/0022-1317-73-12-3141

[9] Okamoto H , Tsuda F , Sakugawa H , et al. Typing hepatitis B virus by homology in nucleotide sequence: comparison of surface antigen subtypes. J Gen Virol. 1988;69(Pt 10):2575‐2583. 3171552 10.1099/0022-1317-69-10-2575

[10] Norder H , Courouce AM , Coursaget P , et al. Genetic diversity of hepatitis B virus strains derived worldwide: genotypes, subgenotypes, and HBsAg subtypes. Intervirology. 2004;47(6):289‐309. 15564741 10.1159/000080872

[11] Yin J , Zhang H , He Y , et al. Distribution and hepatocellular carcinoma‐related viral properties of hepatitis B virus genotypes in Mainland China: a community‐based study. Cancer Epidemiol Biomarkers Prev. 2010;19(3):777‐786. 20160279 10.1158/1055-9965.EPI-09-1001

[12] Milosevic I , Delic D , Lazarevic I , et al. The significance of hepatitis B virus (HBV) genotypes for the disease and treatment outcome among patients with chronic hepatitis B in Serbia. J Clin Virol. 2013;58(1):54‐58. 23838671 10.1016/j.jcv.2013.06.017

[13] Thakur V , Sarin SK , Rehman S , Guptan RC , Kazim SN , Kumar S . Role of HBV genotype in predicting response to lamivudine therapy in patients with chronic hepatitis B. Indian J Gastroenterol. 2005;24(1):12‐15. 15778519

[14] Zhang HW , Yin JH , Li YT , et al. Risk factors for acute hepatitis B and its progression to chronic hepatitis in Shanghai, China. Gut. 2008;57(12):1713‐1720. 18755887 10.1136/gut.2008.157149 PMC2582333

[15] Wen J , Song CI , Jiang D , et al. Hepatitis B virus genotype, mutations, human leukocyte antigen polymorphisms and their interactions in hepatocellular carcinoma: a multi‐centre case‐control study. Sci Rep. 2015;5:16489. 26568165 10.1038/srep16489 PMC4644975

[16] Bai X , Zhu Y , Jin Y , et al. Temporal acquisition of sequential mutations in the enhancer II and basal core promoter of HBV in individuals at high risk for hepatocellular carcinoma. Carcinogenesis. 2011;32(1):63‐68. 20876702 10.1093/carcin/bgq195 PMC3025712

[17] Yuen M‐F , Tanaka Y , Fong D‐T , et al. Independent risk factors and predictive score for the development of hepatocellular carcinoma in chronic hepatitis B. J Hepatol. 2009;50(1):80‐88. 18977053 10.1016/j.jhep.2008.07.023

[18] Yeh C‐T , So M , Ng J , et al. Hepatitis B virus‐DNA level and basal core promoter A1762T/G1764A mutation in liver tissue independently predict postoperative survival in hepatocellular carcinoma. Hepatology. 2010;52(6):1922‐1933. 20814897 10.1002/hep.23898

[19] Xie Y , Liu S , Zhao Y , Zhang L , Liu B , Guo Z . Precore/core region mutations in hepatitis B virus DNA predict postoperative survival in hepatocellular carcinoma. PLoS ONE. 2015;10(7):e0133393. 26208136 10.1371/journal.pone.0133393 PMC4514880

[20] Gaglio P , Singh S , Degertekin B , et al. Impact of the hepatitis B virus genotype on pre‐ and post‐liver transplantation outcomes. Liver Transpl. 2008;14(10):1420‐1427. 18825703 10.1002/lt.21563

[21] Wang C , Lu Y , Chen Y , et al. Prognostic factors and recurrence of hepatitis B‐related hepatocellular carcinoma after argon‐helium cryoablation: a prospective study. Clin Exp Metas. 2009;26(7):839‐848. 10.1007/s10585-009-9283-6 19784786

[22] Xie K , Liu J , Zhu L , et al. A potentially functional polymorphism in the promoter region of let‐7 family is associated with survival of hepatocellular carcinoma. Cancer Epidemiol. 2013;37(6):998‐1002. 24103425 10.1016/j.canep.2013.09.005

[23] Forner A , Llovet JM , Bruix J . Hepatocellular carcinoma. Lancet. 2012;379(9822):1245‐1255. 22353262 10.1016/S0140-6736(11)61347-0 PMC13537732

[24] Kao JH , Chen PJ , Lai MY , Chen DS . Genotypes and clinical phenotypes of hepatitis B virus in patients with chronic hepatitis B virus infection. J Clin Microbiol. 2002;40(4):1207‐1209. 11923332 10.1128/JCM.40.4.1207-1209.2002 PMC140384

[25] Kao JH , Chen PJ , Lai MY , Chen DS . Hepatitis B genotypes correlate with clinical outcomes in patients with chronic hepatitis B. Gastroenterology. 2000;118(3):554‐559. 10702206 10.1016/s0016-5085(00)70261-7

[26] Orito E , Ichida T , Sakugawa H , et al. Geographic distribution of hepatitis B virus (HBV) genotype in patients with chronic HBV infection in Japan. Hepatology. 2001;34(3):590‐594. 11526547 10.1053/jhep.2001.27221

[27] Kao JH , Wu NH , Chen PJ , Lai MY , Chen DS . Hepatitis B genotypes and the response to interferon therapy. J Hepatol. 2000;33(6):998‐1002. 11131465 10.1016/s0168-8278(00)80135-x

[28] Chu CJ , Hussain M , Lok AS . Hepatitis B virus genotype B is associated with earlier HBeAg seroconversion compared with hepatitis B virus genotype C. Gastroenterology. 2002;122(7):1756‐1762. 12055581 10.1053/gast.2002.33588

[29] Liang T‐J , Mok K‐T , Liu S‐I , et al. Hepatitis B genotype C correlated with poor surgical outcomes for hepatocellular carcinoma. J Am Coll Surg. 2010;211(5):580‐586. 20851644 10.1016/j.jamcollsurg.2010.06.020

[30] Chen JD , Liu CJ , Lee PH , et al. Hepatitis B genotypes correlate with tumor recurrence after curative resection of hepatocellular carcinoma. Clin Gastroenterol Hepatol. 2004;2(1):64‐71. 15017634 10.1016/s1542-3565(03)00293-3

[31] Chen C‐H , Lee C‐M , Lu S‐N , et al. Clinical significance of hepatitis B virus (HBV) genotypes and precore and core promoter mutations affecting HBV e antigen expression in Taiwan. J Clin Microbiol. 2005;43(12):6000‐6006. 16333089 10.1128/JCM.43.12.6000-6006.2005 PMC1317177

[32] Jammeh S , Tavner F , Watson R , Thomas HC , Karayiannis P . Effect of basal core promoter and pre‐core mutations on hepatitis B virus replication. J Gen Virol. 2008;89(Pt 4):901‐909. 18343830 10.1099/vir.0.83468-0

[33] Fang Z‐L , Sabin CA , Dong B‐Q , et al. The association of HBV core promoter double mutations (A1762T and G1764A) with viral load differs between HBeAg positive and anti‐HBe positive individuals: a longitudinal analysis. J Hepatol. 2009;50(2):273‐280. 19070921 10.1016/j.jhep.2008.09.014 PMC2648871

[34] Sinn DH , Lee J , Goo J , et al. Hepatocellular carcinoma risk in chronic hepatitis B virus‐infected compensated cirrhosis patients with low viral load. Hepatology. 2015;62(3):694‐701. 25963803 10.1002/hep.27889

[35] Yang YU , Wen F , Li J , et al. A high baseline HBV load and antiviral therapy affect the survival of patients with advanced HBV‐related HCC treated with sorafenib. Liver Int. 2015;35(9):2147‐2154. 25676812 10.1111/liv.12805

[36] Yeo W , Mo F , Chan SL , et al. Hepatitis B viral load predicts survival of HCC patients undergoing systemic chemotherapy. Hepatology. 2007;45(6):1382‐1389. 17539025 10.1002/hep.21572

[37] Domingo E , Sheldon J , Perales C . Viral quasispecies evolution. Microbiol Mol Biol Rev. 2012;76(2):159‐216. 22688811 10.1128/MMBR.05023-11 PMC3372249

